# Healing Effect of *Pistacia Atlantica* Fruit Oil Extract in Acetic Acid-Induced Colitis in Rats

**Published:** 2014-11

**Authors:** Nader Tanideh, Samira Masoumi, Massood Hosseinzadeh, Ali Reza Safarpour, Hoda Erjaee, Omid Koohi-Hosseinabadi, Salar Rahimikazerooni

**Affiliations:** 1 Shiraz Burn Research Center, Colorectal Research Center, Faghihi Hospital, Shiraz University of Medical Sciences, Shiraz, Iran;; 2 Student Research Committee, Shiraz University of Medical Sciences, Shiraz, Iran;; 3 Colorectal Research Center, Faghihi Hospital, Shiraz University of Medical Sciences, Shiraz, Iran;; 4 Student Research Committee, Veterinary School, Shiraz University, Shiraz, Iran;; 5 Center for Comparative and Experimental Medicine, Shiraz University of Medical Sciences, Shiraz, Iran

**Keywords:** Enema, Colitis, Rat

## Abstract

**Background:** Considering the anti-oxidant properties of *Pistacia atlantica *and lack of data regarding its efficacy in the treatment of ulcerative colitis, this study aims at investigating the effect of the *Pistacia atlantica* fruit extract in treating experimentally induced colitis in a rat model.

**Methods:** Seventy male Sprague-Dawley rats (weighing 220±20 g) were used. All rats fasted 24 hours before the experimental procedure. The rats were randomly divided into 7 groups, each containing 10 induced colitis with 2ml acetic acid (3%). Group 1 (Asacol), group 2 (base gel) and group 7 (without treatment) were assigned as control groups. Group 3 (300 mg/ml) and group 4 (600 mg/ml) received *Pistacia atlantica* fruit orally. Group 5 (10% gel) and group 6 (20% gel) received *Pistacia atlantica* in the form of gel as enema. Macroscopic, histopathological examination and MDA measurement were carried out.

**Results:** All groups revealed significant macroscopic healing in comparison with group 7 (P<0.001). Regarding microscopic findings in the treatment groups compared with group 7, the latter group differed significantly with groups 1, 2, 4 and 6 (P<0.001). There was a significant statistical difference in MDA scores of the seven treatment groups (F(5,54)=76.61, P<0.001). Post-hoc comparisons indicated that the mean±SD score of Asacol treated group (1.57±0.045) was not significantly different from groups 4 (1.62±0.024) and 6 *(*1.58±0.028).

**Conclusion: **Our study showed that a high dose of *Pistacia atlantica* fruit oil extract, administered orally and rectally can improve colitis physiologically and pathologically in a rat model, and may be efficient for ulcerative colitis.

## Introduction

Inflammatory bowel disease (IBD) is characterized by chronic, relapsing, idiopathic inflammation of the gastrointestinal tract. The two major forms of IBD are ulcerative colitis and Crohn’s disease.^1^ These two forms of IBD are considered to be more frequent in northern Europe and North America. Although a lower prevalence has been reported in Asia (due to the traditional diet containing a variety of flavonoids which might be a protective factor for colon disease), IBD is on a rising trend.^1^^-^^3^ The exact cause of the disease is unknown, however few possible factors could be genetic, immunologic, environmental and oxidative stress.^2^^,^^3^ Additionally, evidence reveals that reactive oxygen species (ROS) and initiation of lipid peroxidation which are produced in inflamed mucosa, play an important role in the pathogenesis of colitis.^4^ Oxidative stress causes production of free radicals that elevate inflammatory mediators and damage intestinal mucosa.^4^^,^^5^ IBD patients also have low quality of life and risk factor for colon cancer.^6^

Anti-inflammatory agents such as glucocorticoids and salicylates as well as biological agents against tumor necrosis factor-α (TNF-α) are common treatments for ulcerative colitis.^5^ These drugs have various side effects; therefore, there is a rising trend in the world to use herbal drugs for treating ulcerative colitis.^7^ Herbal medicines are widely used in traditional methods of curing diseases among native people. Moreover, in recent years the therapeutic effects of some herbal drugs such as antioxidants are proven in clinical settings.^4^

*Pistacia atlantica* or Bene (from the family Anacardiaceae) is a native fruit in Iran that is used in traditional herbal medicine.^8^ *Pistacia atlantica’s* oil, which is called the Bene Hull Oil (BHO) has been recently introduced to the world as a highly stable compound with anti-oxidative properties. The components of BHO include 6.5% unsaponifiable matter (common in vegetable oil), carotenes, tocopherols and alcohols. Tocopherols and tocotrienols have antioxidant activity and act similar to vitamin E which is beneficial for human health.^9^^,^^10^

*Pistacia atlantica* is believed to have several therapeutic properties such as relieving upper abdominal discomfort and pain, dyspepsia and peptic ulcer. Pistacia spp. is a diuretic and stimulant agents.^11^ Several studies have reported the antimicrobial and analgesic activity of *Pistacia atlantica.*^12^^-^^14^

Antioxidant properties of *Pistacia atlantica* might be an alternative medicine or beneficial food for the prevention or treatment of IBD patients. Considering the lack of data regarding its efficacy in treating ulcerative colitis, this study investigates the effect of the *Pistacia atlantica* fruit extract in treating experimentally induced colitis in a rat model.

## Materials and Methods

*Gel Preparation*

Gel preparation was done in a pharmacology lab under the supervision of expert technicians. The main substance was carboxymethyl cellulose (CMC). 2% sodium CMC was mixed in 5% glycerol and continuously stirred with a mixer at 500 RPM in order to prepare a gel-forming agent. In the next step, *Pistacia atlantica* extracts (10%, and 20%) were added to deionized water. The mixtures were gradually added to the Na-CMC with glycerol and finally the prepared gel was homogenized for 30 minutes and all formulations were collected in an aluminum tube in the refrigerator.

*Animals*

The present animal study was approved by the Ethics Committee of Shiraz University of Medical Sciences (registration number: 90-3894) and all relevant considerations regarding animal rights were taken into account. This study was carried out at the Laboratory Animal Center of Shiraz University of Medical Sciences, Shiraz, Iran. Seventy male Sprague-Dawley rats weighing 220±20g were used. Animals were housed in standard cages with free access to tap water and standard food (ad libitum) and maintained at a controlled temperature (23±1ºC) with a 12/12 hour light-dark cycle. All rats fasted for 24 hours before the experimental procedure. 

*Extract Preparation*

*Pistacia atlantica* fresh fruits were prepared from Shiraz (Fars, Southern Iran) and authenticated by Pharmacology Department of Shiraz Medical School. For the preparation of oil extract, dried and finely powdered fruit (100 g) was soaked in an adequate volume of N-hexane (450 cc) and the extraction was continued for 48 hours to obtain the full extract using the percolation method. The extract was then filtered and evaporated in a rotary evaporator under reduced pressure resulting in a semisolid and oily extract. The final product was then prepared in two doses of 300 mg/kg and 600 mg/kg. The gel form was prepared in concentrations of 10% and 20%.

*Experimental Design*

Colitis was induced in the rats with 2 ml acetic acid 3% by intracolonic instillation through a polyethylene catheter, which was placed 8 cm from the anus under anesthesia with ketamine (90 mg/kg) and xylazine (5 mg/kg). The rats were randomly divided into 7 groups of ten, as follows:

- Groups 1 and 2 were the control groups, respectively receiving 1 ml Asacol (Mesalamine) and 1 ml base gel (carboxymethylcellulose) as enema.
- Groups 3 and 4 received 300 and 600 mg/kg *Pistacia atlantica* fruit oil extract orally, respectively.
- Groups 5 and 6 received 10% and 20% gel from *Pistacia atlantica’s* fruit as enema.
- Group 7 was the third control group in which colitis were induced without treatment.

All groups were subjected to relevant protocol and after 7 days, rats were sacrificed in a CO_2_ chamber. The abdomen was opened and the colon was exposed. The distal 8 cm of the colon was removed and opened by longitudinal incision. The mucosal surface was washed with saline buffer and then the mucosal injury was macroscopically assessed using Morris et al.’s^15^ grading scale (0: no change, 1: mucosal erythema only, 2: mild mucosal edema, slight bleeding or slight erosion, 3: moderate edema, bleeding ulcers or erosions, 4: severe ulceration, erosions, edema and tissue necrosis).

For histological examination, the colon tissue was fixed in 10% formalin sectioned in 5 μm thick sections, and stained with haematoxylin and eosin. All slides were reviewed by a single blinded pathologist and sub-classified according to the “repair scoring system”.^16^ Inflammation and crypt damage was assessed and the sections were coded using a modified validated scoring scheme as described by Appleyard and Wallace.^17^

*Tissue MDA Measurements*

MDA production was measured in tissue samples by the method proposed by Ohkawa et al.^18^ MDA forms a colored complex in the presence of thiobarbituric acid (TBA) and n-butanol, which is detectable by the measurement of absorbance at 532 nm. Absorbance was measured using a Shimadzu UV-160 spectrophotometer. 1,1´,3,3´-tetramethoxypropane was used as standard and the results were expressed as µmol/g protein.^19^

*Statistical Analysis*

Statistical analyses were performed using SPSS 21 software (SPSS, Chicago, IL, USA). One way ANOVA, Kruskal-Wallis and Mann-Whitney tests were used. Data were expressed as mean±SD, frequency, and percentages. A P value <0.05 was considered as statistically significant. Moreover, Bonferroni correction was performed as an appropriate form of analysis. 

## Results

*Macroscopic Findings*

All specimens were assessed according to the Moris scoring system.^15^ Kruskal–Wallis test revealed significant macroscopic healing between group 7 (colitis induced without treatment) and other groups (P<0.001).

To determine the exact difference between the groups, Mann-Whitney U test between pairs of groups was performed. A statistically significant difference was seen between groups 7 and 1 (standard treatment with Asacol) (P<0.001), groups 7 and 4 (extract 600 mg oral) (P=0.002) and groups 7 and 6 (extract 20% gel) (P=0.001). The Bonferroni correction method was applied according to the number of comparisons.

*Histopathological* *Findings*

**Figure 1:**
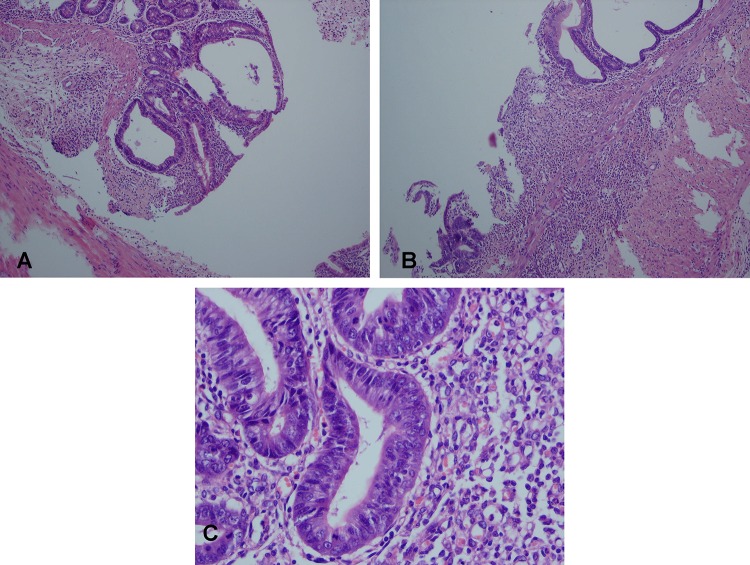
Photos from the pathology slides showing the effects of the Pistacia atlantica. A: Focal crypt disorganization and cryptitis (group 6, H&E, ×400), B: Ulceration, moderate colitis, and crypt disorganization (groups 5 and 3, H&E, ×100), C: Regeneration, granulation tissue and mild inflammation (groups 6 and 4, H&E, ×400

Microscopic findings included focal crypt disorganization, ulceration, moderate colitis, and crypt disorganization, regeneration, granulation tissue and mild inflammation (figure 1). The samples were classified microscopically based on the repair scoring system.^16^ With respect to the microscopic findings in the treatment groups compared with group 7 (colitis induced without treatment), as shown in table 1, this group differed significantly from groups 1, 4 and 6 (P<0.001). To control for the type 1 error, the Bonferroni correction method was applied to the alpha value.

**Table 1:** Microscopic and macroscopic feature scores in the seven studied groups to evaluate the effect of *Pistacia atlantica* in acetic acid-induced colitis in rats

**Groups**	**Macroscopic scores** **(mean** **±** **SD)**	**Histopathologic** ** scores** **(mean** **±** **SD)**
Control (Asacol)	1±0	1±0
Colitis (non-treated)	3.88±0.75	3±1.67
Base of gel	2.10±0.91	2±1.40
Enema 20% gel	1.20±1.03	1.20±1.07
Enema 10% gel	1.50±0.97	1.60±1.42
Oral 300 mg	2.60±1.14	2.80±1.87
Oral 600 mg	1.37±1.30	0.87±1.12

* *

*MDA Test Comparison*

**Figure 2:**
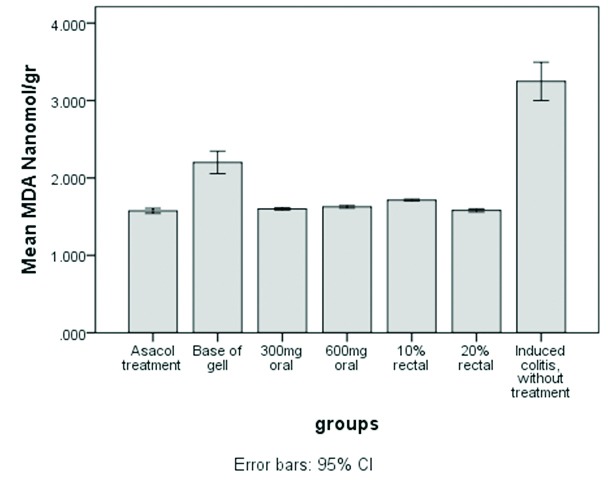
Comparison of mean concentration of MDA in seven study groups (nmol/g) to evaluate the effect of Pistacia atlantica in acetic acid-induced colitis in rats.

A one-way between-group analysis of variance was used to analyze the impact of the new treatment on inflammation levels, as measured by the MDA test. There was a statistically significant difference in MDA scores of the seven groups (F (5,54)=76.61, P<0.001). Post-hoc comparisons using the LSD test indicated that the mean±SD score for group 1 (1.57±0.045) was not significantly different from groups 4 (1.62±0.024) and 6 (1.58±0.028) as shown in figure 2. This result means probable same anti-inflammatory effects of both high doses of the extract (600 mg orally and 20% gel) in comparison with Asacol.

## Discussion

In this study, the effect of the *Pistacia atlantica* fruit extract as well as its effect on reducing inflammation and contribution to tissue repair was examined in acetic acid induced colitis in a rat model. Intrarectal acetic acid injection is an easy, repeatable, and cost-effective method for experimental colitis on small laboratory animals. It is comparable with inflammation and ulcers in the epithelium of the colon, and findings such as damaging the crypts of the intestine and killing epithelial cells and lowering goblet cells. These changes are similar to ulcerative colitis.^19^

The findings of this investigation demonstrated that *Pistacia atlantica* fruit oil extract reduces colonic injury by suppressing oxidative damage. The pathogenesis of the disease was assessed by evaluating different parameters such as macroscopic score, microscopic score and oxidative stress markers like MDA level. We found an improvement and lower MDA levels in the groups treated with the high dose of fruit extract compared with the untreated group. Histological and macroscopic data confirmed that both oral and rectal administration of *Pistacia atlantica* fruit oil extract could alleviate bowel inflammation. Asacole was used as the standard drug, which produced no significant difference in the above parameters in comparison with *Pistacia atlantica* at doses 600 mg/kg (orally) and 20% gel form (rectally).

The fruits of *Pistacia atlantica* were found to be rich in proteins, oils, fibers, and unsaturated fatty acids, suggesting that they may be valuable as food. The major components of *Pistacia atlantica* are α-tocopherols and important sterols that have antioxidant properties and health benefits such as being precursors of vitamin D and lowering blood cholesterol.^20^ Their antioxidant activity is attributed to the presence of tocopherols and tocotrienols, and are as active as vitamin E.^1^^,^^2^ Shimizu et al.,^21^ showed that a vitamin E enriched diet could be beneficial in the treatment of ulcerative colitis in rats. The therapeutic role of *Pistacia atlantica* for ulcerative colitis in rats may be similar to vitamin E, which is consistent with findings by Shimizu et al.^21^

Ademoglu et al.^22^ found that vitamin E and selenium supplementation, significantly reduced both the severity of colonic lesions and the levels of MDA and protein carbonyl. The researchers offered antioxidants and some micronutrients as therapeutic agents. In another study, vitamin E levels were also found to be low in some patients with IBD and low levels of vitamin E was found during active disease.^23^

Vitamin E has beneficial effects intraluminally. It is a lipid soluble radical scavenger and a lipid peroxidation suppressor, which reduces the free radical generating capacity of the feces in ulcerative colitis.^24^^,^^25^ Vitamin E can also reduce intestinal inflammation by preventing the activation of the transcription factor NF-κB, which plays an important role in intestinal inflammation.^26^^,^^27^ This likelihood is supported by several in vitro studies in which vitamin E inhibited tumor necrosis factor-α-induced NF-κB activation in human Jurkat T-cells^28^ and inhibited the activation and translocation of NF-κB to the nucleus and the binding of the activated proteins to the κB DNA site.^29^

Dost et al.^4^ found that *Hypericum perforatum* is effective in decreasing MDA levels in TNBS-induced colitis in rats due to its antioxidant activity. Consistently, in our study, MDA levels decreased in the treatment groups and this reduction may be attributed to the protective effect of *Pistacia atlantica* against lipid peroxidation, which may in turn be due to the presence of tochpherols and tocotrienols.

In another study, CMC-treated mice had significantly lower histology scores and lower colon weight/length ratios, indicating that CMC-treatment alone may play a role in reducing colonic injury.^30^ CMC appears to be beneficial despite high levels of pro-inflammatory cytokines, suggesting a mechanism of action independent of an imbalance in cytokine production. Possible mechanisms could include an increase in cellulose derived nutrients to promote mucosal healing or immune function as well as variations in the intestinal flora. Thus, CMC may have exerted its positive effects on the intestinal lumen by increasing the viscosity of the mucous layer, thereby altering patterns of pathogenic bacteria migration through the mucous layer, where they would have otherwise led to inflammation and damage to the intestine.^29^

Common belief indicates that herbal therapies are safer and less toxic than conventional treatment in patients with IBD. Herbal medicines such as Curcumin, Aloe Vera, and *Boswellia Serrata* are effective in ulcerative colitis based on their antioxidant properties, which is similar to that of *Pestacia atlantica.*^31^ However, not all studies have shown consistent and promising results with respect to the role of herbal therapy in IBD. The reasons for such controversial findings could be related to different study designs, small sample size, lack of adequate control, and the variation in drug formulations and doses.^31^ All these confounding factors were considered in this study.

Most of the herbal therapies used for IBD have been associated with minimal adverse effects compared with glycocorticosteroids. According to previous studies, *Pistacia atlantica*, also has no proven hepatotoxicity effects.^32^^,^^33^ This treatment will be attractive to many patients and cost effective in comparison with glycocorticoid therapy. No toxic effect of *Pistacia atlantica* has been reported, showing that higher doses of this extract may be effective and safe. Recently, resistance to drugs, their toxicity and side effects have been reported; therefore, there is a great need for the development of an effective, safe and topical herbal drugs such as a *Pistacia atlantica* extract for different forms of ulcerative colitis.

This study had limitations such as the lack of evaluation of anti-inflammatory markers and stereology of colon tissue. Certainly, further studies are required to clarify the mechanisms by which *Pistacia atlantica* exerts its beneficial effects and its efficacy should be confirmed through clinical trials. This study opens a path for future clinical trials in humans.

## Conclusion

*Pistacia atlantica* fruit extract orally and rectally can improve induced colitis physiologically and pathologically in a rat model. This therapy is potentially safe, cost-effective, and efficient for treating ulcerative colitis. Further investigations of its clinical application in human model are warranted.
